# Profiling of Bacterial Communities of Hospital Wastewater Reveals Clinically Relevant Genera and Antimicrobial Resistance Genes

**DOI:** 10.3390/microorganisms13061316

**Published:** 2025-06-05

**Authors:** Clemente Cruz-Cruz, Javier Gaytán-Cervantes, Carolina González-Torres, Andres Emmanuel Nolasco-Rojas, Miguel Ángel Loyola-Cruz, Laura Delgado-Balbuena, Josué Delgado-Balbuena, Marianela Paredes-Mendoza, María Concepción Tamayo-Ordóñez, Yahaira de Jesús Tamayo-Ordoñez, Emilio Mariano Durán-Manuel, Araceli Rojas-Bernabé, Carlos Alberto Jiménez-Zamarripa, Oscar Sosa-Hernández, Omar Agni García-Hernández, Esther Ocharan-Hernández, Paola Berenice Zárate-Segura, Elizabeth González-Terreros, Daniel Alejandro Ramírez-Villanueva, Claudia Camelia Calzada-Mendoza, Juan Manuel Bello-López

**Affiliations:** 1 Hospital Juárez de México, Mexico City 07760, Mexico; 2 Sección de Estudios de Posgrado e Investigación, Escuela Superior de Medicina, Instituto Politécnico Nacional, Mexico City 11340, Mexico; 3 División de Desarrollo de la Investigación en Salud, Centro Médico Nacional Siglo XXI, Mexico City 06720, Mexico; 4 Centro Nacional de Investigación Disciplinaria Agricultura Familiar, Km 8.5, Carretera Ojuelos-Lagos de Moreno, Jalisco 47540, Mexico; 5 División de Tecnología Ambiental, Universidad Tecnológica de Nezahualcóyotl, Nezahualcóyotl 57000, Mexico; 6 Facultad de Ciencias Químicas, Universidad Autónoma de Coahuila, Saltillo 25280, Mexico; 7 Centro de Biotecnología Genómica, Instituto Politécnico Nacional, Reynosa 88710, Mexico; 8 Hospital Psiquiátrico Dr. Samuel Ramírez Moreno, Valle de Chalco Solidaridad 56619, Mexico; 9 Facultad de Medicina, Universidad Nacional Autónoma de México, Mexico City 54090, Mexico; 10 Instituto de Estudios Ambientales, Universidad de la Sierra Juárez, Ixtlán de Juárez Oaxaca 68725, Mexico

**Keywords:** hospital wastewater, bacteria, antimicrobial resistance, V3-V4 hypervariable

## Abstract

In Mexico, hospital wastewater (HWW) is a source of chemical and microbiological contamination, and it is released into the municipal sewage system without prior treatment. This water may contain pathogenic bacteria and antimicrobial resistance genes, which represent a risk to Public Health and the environment. So far, there are no studies that analyse this problem comprehensively, relating bacterial population structures, chemical contaminants, and seasonality. The aim of this work was to seasonally characterise the bacterial communities of HWW, including clinically relevant bacteria and resistance genes in Hospital Juárez de México (HJM), and to evaluate the impact of physicochemical factors on their composition. A one-year observational, cross-sectional study was conducted at five HWW discharge points of HJM. Fourteen physicochemical parameters were determined by using standard methodologies, and statistical differences between discharges and seasons were evaluated. Bacterial communities were analysed by targeted amplicon sequencing of the V3-V4 region of the *16S rRNA* gene. In addition, the presence of eight antimicrobial resistance genes of local epidemiological importance was assessed. Data were analysed using alpha and beta diversity indices, principal component analysis, and multivariate statistical tests. HWW showed high taxonomic diversity, with Proteobacteria, Firmicutes, and Bacteroidetes standing out. Clinically relevant bacteria were identified in 73.3% of the analyses, with *Enterobacter* and *Escherichia-Shigella* predominating. Total and dissolved solids, temperature, nitrate, and pH significantly influenced the bacterial composition of HWW. Seven out of the eight genes evaluated were identified, with *bla_KPC_*, *bla_OXA-40_*, and *mcr-1* being the most frequent, showing significant seasonal differences. This study underlines the microbiological and chemical complexity of HWW, highlighting the impact of clinically relevant bacteria and antimicrobial resistance genes on Public Health. The findings emphasise the need to implement hospital waste management programmes and ideally specific treatment plants to minimise the associated risks and protect the environment and human health.

## 1. Introduction

Healthcare facilities release large amounts of hospital wastewater (HWW) into the municipal sewer system; the volume of HWW released depends on several factors, such as the geographical location of the hospital, the type and capacity of care, medical services and procedures performed, hygiene practices for infection control, healthcare infrastructure and technology, and local policies and/or regulations, among others [1,2,3,4]. For example, HJM discharges approximately 150 m^3^ of untreated wastewater per day, which represents only ~0.004% of the total daily volume processed by Mexico City’s municipal sewage system [5]. However, despite this relatively small contribution by volume, hospital effluent is known to carry a disproportionately high load of chemical and microbiological contaminants. Most hospitals in Mexico, including HJM, discharge their effluent directly into the municipal system without any pretreatment. Although some may have grease traps or sedimentation chambers, these are not designed to reduce the microbial load. Recent studies in Mexico have documented the presence of multidrug-resistant bacteria and antimicrobial resistance genes (ARGs) in municipal sewage and HWW both before and after treatment [6,7]. Conversely, due to the inadequate management of HWWs, it leads to these waters being released into the municipal sewage system without prior treatment and dangerously contaminated by a wide diversity of pathogens such as viruses, bacteria, fungus, parasites, and chemical pollutants that can affect the environment and human health [8,9]. The direct implications of HWW are that they mainly contaminate aquatic ecosystems, being one of the issues of greatest environmental attention due to the impacts on aquatic life and human health through direct or indirect contact [10].

A relevant aspect is that due to the COVID-19 pandemic, attention was drawn to active microbiological surveillance of HWW released from hospital facilities as they have been recognised as reservoirs of biological contaminants, including the new coronavirus SARS-CoV-2, a causative agent of COVID-19 and bacteria carrying antimicrobial resistant determinants [6,11,12,13,14]. In this context, HWW may contain a high load of microbiological and anthropogenic contaminants, also called macro-pollutants, where carbapenem-resistant pathogenic ESKAPE bacteria are the most important [15]. These HWWs with ESKAPE bacteria endanger human health and the environment, creating a biological imbalance, thus constituting a major Public Health problem. It is important to mention that Mexico has no legislation regulating HWWs; i.e., they are released without prior treatment into the municipal sewage system. This is why there is little or no concern in this regard, so much so that, to our knowledge, there are few Mexican research studies that address the study of this problem [7,14,16]. Although some studies used conventional culture-based approaches, they reported the presence of multidrug-resistant bacteria, including *Staphylococcus* sp., *Enterococcus* sp., *K. pneumoniae*, *E. coli*, and *A. baumannii*, as well as the detection of carbapenemase genes such as *bla_NDM_*, *bla_OXA-48_*, and *bla_KPC_*. These findings, although based on classical microbiology, support the relevance of studying HWW as a source of antimicrobial resistance dissemination in Mexico.

In contrast, massive sequencing tools have revolutionised the analysis of bacterial communities by allowing the identification and characterisation of all populations using two important parameters in microbiota studies: taxonomic diversity and relative abundance. This approach also offers a comprehensive view of the interactions between microorganisms and their environment, including other macro-pollutants such as acidity, alkalinity, hardness, solid loading, N$O_{3}^{-}$, and P$O_{4}^{-2}$, among others, and assesses how they influence the structure and dynamics of bacterial communities on a seasonal basis. For this reason, current microbiological research in our working group is not only restricted to problems in the hospital environment but also to those that may have an impact on the environment and the population, and there is no better study model than the HWW of Hospital Juárez de México (HJM), a tertiary hospital located in the north of Mexico City. This is why the profiling of HWW bacterial communities though massive sequencing of the *16S rRNA* gene (V3-V4 hypervariable region) is essential to understand the complexity of microbiological contamination, the seasonal dynamics of its release, and the influence of other hospitals’ macro-pollutants on these populations. The aim of this work was to seasonally characterise the bacterial communities released (including clinically relevant bacterial genus) through the discharge points of the HWW of HJM to evaluate the impact of other macro-pollutants as modulators on their composition and to understand the bacterial diversity and antimicrobial resistance genes in HWW through massive sequencing of the *16S rRNA* gene and end-point PCR, considering the epidemiology of HJM and other antimicrobial resistance mechanisms that may be present (resistance to polymyxins due to the *mcr-1* gene). Environmental and human health implications of the release of clinically important microbiological contamination through HWW are analysed and discussed.

## 2. Materials and Methods

### 2.1. Water Consumption and HWW Discharge Points of HJM

**Figure 1:**
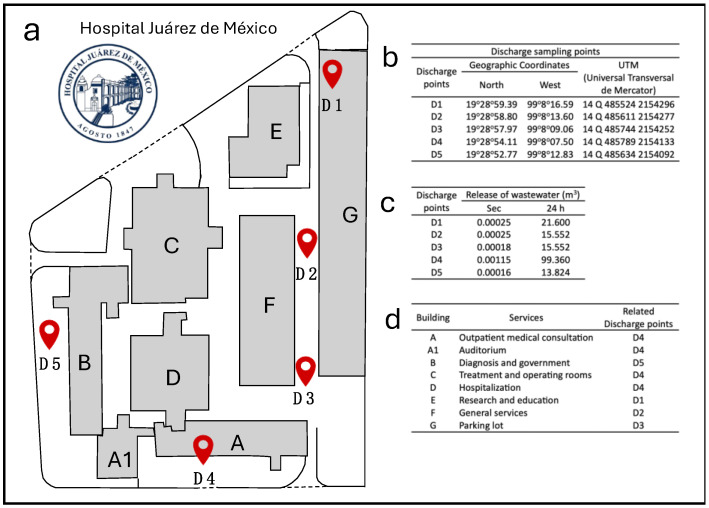
(**a**) Aerial map of HJM with the discharge points (D1 to D5) of HWW; (**b**). Geographical coordinates of their location; (**c**) volume of HWW released in m^3^ per discharge (per second and 24 h); (**d**) hospital services related to each of the discharge sites. The discharge points (D1 to D5) are indicated by the red marking symbol.

Hospital Juárez de México is a tertiary public healthcare institution located in the north of Mexico City, with an annual average of 30,000 patients. It has 391 census beds with an average consumption of 385 L of water by each patient. According to these characteristics, an average HWW discharge of 150 m^3^ per day is generated. Hospital wastewater discharges are released without prior treatment to the municipal public sewerage system of Mexico City through 5 discharge points (D1 to D5) (Figure 1). The HWW from HJM is discharged to nearby receiving water bodies and wastewater treatment plants. Figure 1 shows the aerial map of HJM with the HWW discharge points (D1 to D5), the geographical coordinates of their location, the volume of HWW released in m^3^ per discharge (per second and 24 h), and the hospital services related to each of the discharge sites.

### 2.2. HWW Sample Collection and In Situ Temperature Determination

This study was observational and descriptive from January to December 2024; it was distributed into 24 biweekly monitoring intervals (two per month/per discharge point) with a total of 120 HWW samples collected. For the physicochemical and *16S rRNA* gene massive sequencing analysis of HWW samples, from each discharge site (D1 to D5), 1.5 L of the HWW samples was collected in sterile amber glass bottles in accordance with the Mexican Standard that establishes the characteristics of sampling of residual waters [17]. Several aspects were considered for the standardised collection of HWW samples such as the date (every 1st and 16th day of each month) and fixed morning hours (10:00 a.m.), which are considered to be the busiest time of the day. In situ temperature determination was performed at the time of HWW sample collection [18]. Finally, the collection of all HWW samples was under strict adherence to the biosafety conditions established by the Mexican Standard NMX-AA-003-1980, and all samples were transported at 4 °C to the microbiology laboratory for analysis. Hospital wastewater samples used in this study were collected from the same hospital locations and analysed in the same manner as a previously published study [19]. However, the present work addresses a distinct research objective. In contrast, the previous study employed culture methods to identify specific antimicrobial-resistant pathogens and resistance genes. No results, figures, or sequence data from the previous publication are reused here. This manuscript presents a new dataset and independent analysis focused on taxonomic profiling and its seasonal variation.

### 2.3. Determination of Physicochemical Parameters of HWW

For the determination of the physicochemical parameters of HWW, standardised methodologies based on the Mexican Official Standards for wastewater and domestical wastewater were used [20,21,22,23,24,25,26,27,28,29]. For this purpose, fourteen physicochemical determinations were performed (in triplicate) and grouped into six categories according to their purpose. These determinations were standard quality parameters (temperature, residual chlorine, and pH), chemical equilibrium (acidity, alkalinity, and hardness), electrochemical (electrical conductivity), solid loading (total, suspended, and dissolved), oxidation and organic quality (dissolved O_2_ and chemical O_2_ demand (CO_2_D)), and macronutrients (N$O_{3}^{-}$ and P$O_{4}^{-2}$). Significant differences between the physicochemical factors by discharge were analysed and evaluated by using ANOVA and Tukey’s post hoc test for the fourteen psychochemical parameters in the study period. Significant differences between the discharges (D1 to D5) were established when the *p*-value was <0.05. Additionally, the SPSS v.27.0.1.0 and XLSTAT 2023 statistical software programmes were used for the analysis and graphical representation.

### 2.4. Metagenomic DNA Extraction and Quality Control

DNA metagenomic extraction was performed (per triplicate) according to methods by Chung et al. (2000), with minor modifications as follows [30]: Aliquots of 25 mL of each HWW sample were centrifuged at 5000× *g* for 30 min at 4 °C, and the supernatant was decanted. Pellets were subjected to metagenomic DNA extraction and purification. One millilitre of the EC lysis solution was added to the pellet, resuspended, and incubated at 37 °C for 5 h. For this purpose, the suspension was centrifuged at 13,000 rpm for 5 min, and the supernatant was decanted. A total of 1 ml of the ESP solution was added, and the mixture was incubated at 56 °C overnight.

The pretreatment samples were subjected to metagenomic DNA extraction with the commercial Favorgen^®^ Genomic DNA Kit (Omega BIO-TEC, GA, SA) according to the manufacturer’s instructions. Replicates of metagenomic DNA were pooled (per sample) and quantified by fluorometry using the Qubit 4 Fluorometer (Thermo Fisher Scientific, Waltham, MA, USA). Additionally, they were visualised on horizontal 0.8% agarose gels and subjected to end-point PCR assays using 27F/1492R primers (V1-V9 regions) to determine if DNA samples were amplifiable for the complete *16S rRNA* housekeeping gene (1492 bp) [31]. The V3-V4 hypervariable region of the *16S rRNA* gene was subjected to massive sequencing by using the Illumina platform. Finally, for the *16S rRNA* gene massive sequencing analysis of the HWW samples, water samples from three different origins were included: DW: domestic wastewater (*n* = 6), TW: treated wastewater (*n* = 5), and WW: water well from the Cutzamala and Nezahualcóyotl system of the State of Mexico (*n* = 26).

### 2.5. Targeted Amplicon Sequencing (V3-V4 Hypervariable Region) with the Illumina Platform

The V3-V4 hypervariable region of the *16S rRNA* gene was amplified by PCR using a GoTaq^®^ Green Master Mix (Promega, Madison, WI, USA) and the 341F/805R primers previously reported by Klindworth et al. (2013) [32]. Library preparation was performed according to the Illumina 16S amplicon sequencing protocol. Briefly, *16S rRNA* amplicons (V3-V4 region) were purified with the DNA clean and concentrator kit (Zymo Research, Irvine, CA, USA). Dual indices and Illumina sequencing adapters were attached in a second PCR step by using the Nextera XT Index kit V2 (Illumina, San Diego, CA, USA). Finally, amplicons were purified by using AMPure XP beads (Beckman Coulter, Brea, CA, USA), pooled in equimolar concentrations, and sequenced on a MiSeq Illumina instrument.

### 2.6. Quality Control of Sequencing Data

Paired sequencing FASTQ files were inspected for *Phred* quality scores and adapter content by using FastQC v0.11.9 [33]. The DADA2 v1.20.0 pipeline was used to filter out low-quality reads, perform dereplication, remove chimeric sequences (<2%), and join paired reads [34]. A sequence-by-base analysis was conducted to evaluate quality intervals across base positions in the FASTQ files. Additionally, 16S sequences associated with chloroplasts or mitochondria were removed.

### 2.7. Assignment of Taxonomic Levels and Relative Abundance

Illumina raw sequences [1,999,975 forward sequences: HWW (1,547,989), WW (306,423), DW (108,611), and TW (36,952)] were analysed under the QIIME2 command line (version 2021.11) [35]. Following quality control, the processed sequences were separated and analysed by using the SILVA NR99 database (version 138.1), which contains 510,984 sequences, to achieve phylum-to-species-level annotation [36]. For this purpose, the sequences were grouped according to their origin (HWW, WW, DW, and TW). The taxonomic assignment was conducted by using the R package *phyloseq*, allowing classification at five taxonomic levels (phylum, class, order, family, and genus) [37]. Finally, alluvial diagrams were generated in RAWGraph for clinically relevant bacterial genus liberation by discharge points (D1 to D5), the season of the year, and antibiotic resistance genes [38].

### 2.8. Diversity Analysis

Alpha diversity analysis was performed for the four water sources (HWW, WW, DW, and TW) using the microbiome and *phyloseq* libraries in R. To assess alpha diversity, the following indices were calculated: Chao1 (total species richness), Pielou’s evenness (the evenness of species distribution), Fisher (species richness and relative abundance), Gini–Simpson (probability that two randomly selected sequences belong to different species), inverse Simpson (the effective number of dominant species), and the Shannon index (balance between richness and evenness). To assess the degree to which microbial diversity varies between groups (HWW, WW, DW, and TW), the ANOVA test was applied to determine if there were significant differences in the alpha diversity indices performed (*p* < 0.05). The beta diversity analysis was performed to observe the distribution according to the bacterial composition of the water samples based on their similarities. For this purpose, the weighted UniFrac and unweighted UniFrac method coupled to a principal coordinate analysis (PCoA) was used to visualise the variation between microbial communities. Finally, a PERMANOVA analysis was performed to assess whether differences in microbial composition between groups were statistically significant at *p* < 0.05 [39].

### 2.9. Influence of Physicochemical Factors on Bacterial Communities in HWW

To assess the influence of the assessed physicochemical factors on the bacterial communities in the HWW, a biplot analysis based on principal component analysis (PCA) was performed. This analysis was performed with the purpose of identifying the underlying physicochemical factors that could explain the variability of the bacterial communities as a function of the measured parameters. The analyses were performed by using the ggbiplot (version 0.55) and vegan (version 2.6-4) libraries in R and applying normalisation to the data to ensure comparability between variables. For this purpose, two categorical variables were considered: (A) month: “january to december” and (B) season of the year: “spring, summer, autumn, and winter”. This analysis allowed us to explore possible temporal and seasonal patterns and the influence of physicochemical factors on bacterial communities. The proportions of variability explained by the first two principal components (PC1 and PC2) were 20.9% and 13.5%, respectively.

### 2.10. Detection of Carbapenems and Polymyxin Resistance Genes

End-point PCR assays were performed to detect metallo-β-lactamases (*bla_NDM_*, *bla_VIM_*, and *bla_IMP_*), serine β-lactamases (*bla_KPC_*, *bla_OXA-48_*, *bla_OXA-23_*, and *bla_OXA-40_*), and polymyxins resistance (*mcr-1*) genes using the primers described in Table 1. Amplicons were run in 1 × TBE buffer (pH 8.3), separated via horizontal electrophoresis in 2.0% agarose gels, visualised, compared with an appropriate molecular weight marker, and photographed under UV illumination. Positive controls were obtained from Cortés-Ortíz et al. (2021), Loyola-Cruz et al. (2023), and Cureño-Díaz et al. (2024) [40,41,42]. In the case of the *mcr-1* gene, 10% of the PCR products were sequenced and compared with the nucleotide sequence database (GenBank) by using the BlastX algorithm with strict filter parameters, namely more than 99% nucleotide identity and at least 80% query coverage.

## 3. Results

### 3.1. Evaluation of Physicochemical Parameters of HWW

**Figure 2:**
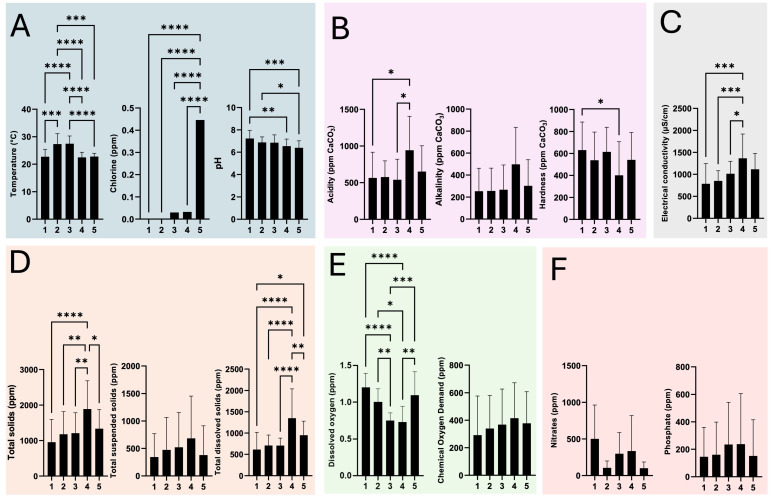
Physicochemical parameters of HWW from HJM. (**A**) Standard quality parameters (temperature, chlorine, and pH), (**B**) chemical equilibrium (acidity, alkalinity, and hardness), (**C**) electrochemical (electrical conductivity), (**D**) solid loading (total, suspended, and dissolved), (**E**) oxidation and organic quality (dissolved O_2_ and chemical O_2_ demand (CO_2_D)), and (**F**) macronutrients (NO^−3^ and P$O_{4}^{-2}$). X-axis: 1–5: Discharge 1 to 5. Significance level: * (0.01), ** (0.001), *** (0.0001), and **** (0.00001).

Figure 2A–F show the results of the fourteen physicochemical factors of the HWW at the five discharge points (D1 to D5) of HJM during one year of monitoring. Overall, the ANOVA and Tukey’s post hoc analysis demonstrated significant differences with varying degrees of statistical significance between the five discharges for ten of the fourteen physicochemical determinations. For the first group of determinations (standard water quality), it was observed that temperature and chlorine showed a higher degree of significance between discharges (*p* = 0.0001 and 0.00001) compared to pH (*p* = 0.01 to 0.001) (Figure 2A). In the case of the pH parameter, a higher degree of significance was observed between D1 vs. D5, while a lower degree of significance was seen between D2 vs. D5, with D1 and D5 being pH neutral and acidic, respectively. Determinations corresponding to chemical equilibrium parameters (Figure 2B) revealed the presence of strong acids, bicarbonates (HC$O_{3}^{-}$), and carbonates (C$O_{3}^{-2}$) in all discharges analysed; however, in contrast, D4 vs. D1 and D3 showed significant differences (*p* = 0.01) in the release of strong acids. Interestingly, D4 showed higher acidity and alkalinity during the study period. With respect to hardness, the only significant difference was identified between D1 vs. D4 in the release of dissolved mineral (Ca^2+^), with the average minimum and maximum values of this mineral being 401 and 630 ppm CaCO_3_, respectively (Figure 2B). The electrical conductivity of the HWW showed that D4 was the one with significant differences (*p* = 0.01 to *p* = 0.0001) compared to the other discharge points (except for D5), with an average maximum value of 1364 mS/cm (Figure 2C). Interestingly, the pH, acidity and alkalinity of discharge point D4 were significantly elevated, which agrees with the maximum electrical conductivity values described above. Determinations pertaining to the quantification of solids loading in the HWW revealed that D4 showed the highest levels for all three determinations (total, suspended, and dissolved solids) compared to the other sampling sites (Figure 2D). The hypoxic conditions due to organic contamination were determined through the quantification of oxidation and organic quality parameters (dissolved O_2_ and COD_2_). The results showed a typical behaviour, where the variable “dissolved O_2_”, although showing significant differences between each discharge, showed an inverse relationship with the variable “CO_2_D” (Figure 2E). Finally, the determination of macronutrients that give rise to aquatic eutrophication revealed the active release of NO^−3^ and P$O_{4}^{-2}$ with no significant differences between each of the discharges (Figure 2F).

### 3.2. Quality Control and Error Models in Sequencing Data

The DADA2 analysis for the quality control of the sequencing data of the ribosomal libraries showed that most of the reads were of good quality above the *phred* quality score (a score of 30). Conversely, a parametric error model was run by using the DADA2 algorithm to determine base transitions. The error model constructed was considered adequate; therefore, we proceeded to the analysis of the taxonomic assignment and relative abundance.

### 3.3. Characteristics of the Bacterial Communities of the HWW, DW, TW, and WW

**Figure 3:**
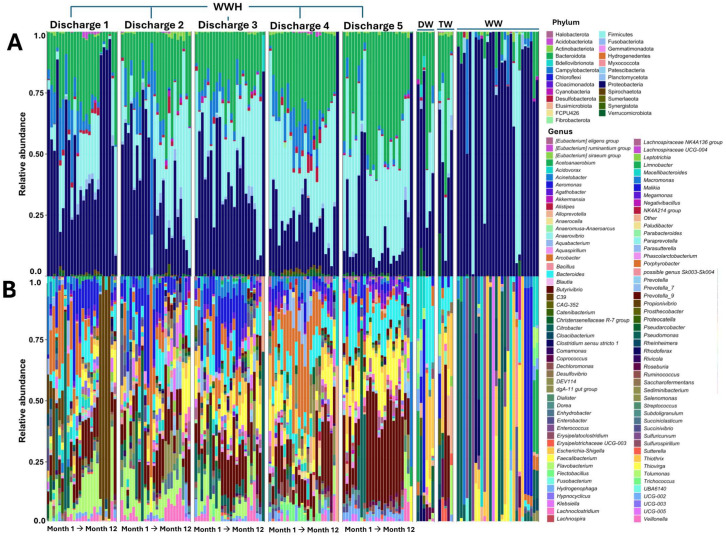
Variation in taxonomic diversity and annual relative abundance (at the phylum and genus levels) of bacterial communities released through the HWW of HJM: (**A**) phylum level and (**B**) genus level. HWW: hospital wastewater, DW: domestic wastewater, TW: treated wastewater, and WW: water well of the Cutzamala and Nezahualcóyotl system.

The seasonal variation (month 1 to month 12) of the annual taxonomic diversity and relative abundance of the bacterial communities released from HWW classified by discharge (D1 to D5) is shown in Figure 3.

For this purpose, a longitudinal analysis of each discharge point was performed while considering two aspects: taxonomic diversity (at the phylum and genus levels) and relative abundance. Finally, to contrast the findings of the HWW, water samples from other origins (from a single intake and time) were included, as they have different microbiological and anthropogenic characteristics (DW, TW, and WW). The results of this analysis showed 25 phyla distributed in all HWW samples, where 3 of them were predominant (Proteobacteria, Bacteroidetes, and Firmicutes), being in D1. Proteobacteria and Firmicutes were the most and least abundant phyla, respectively (Figure 3A). In the case of the taxonomic composition of the other water sources, it was observed that Proteobacteria were predominant compared to HWW, where in some cases, in the WW samples, it was 100% in relative abundance. In the case of DW and TW samples, the three phyla described above were present in similar relative abundances with the predominance of Proteobacteria and Bacteroidetes. For the analysis at the genus taxonomic level of the HWW, DW, TW, and WW, the top-100 list of predominant genera was considered. The results revealed that, within these genera, eight genera were found to harbour the highest number of species identified in the HWW samples. The predominant genera with the highest number of species were *Bacteroides* (*n* = 13), *Acinetobacter* (*n* = 9), *Pseudomonas* (*n* = 8), *Aeromonas* (*n* = 8), *Enterobacter* (*n* = 4), *Flavobacterium* (*n* = 4), *Klebsiella* (*n* = 3), and *Prevotella* (*n* = 3) (Figure 3B).

The graphs of the taxonomic composition and relative abundance at the remaining three taxonomic levels of the HWW, DW, TW, and WW are deposited in the Supplementary Material. A high number of bacterial genera were observed, such as *Arcobacter*, *Feacalibacterium*, *Escherichia-Shigella*, and *Propionivibrio*, and were identified with high relative abundances. In some cases, such as *Propionivibrio*, relative abundances reached nearly 100% in D1 (last two months of the year). An emergent bacterial genus of medical interest that showed important relative abundances during the study period in the five discharges (with predominance in D1 and D3) was *Aeromonas* spp. The presence of strict anaerobes of the genus *Prevotella* was present in all discharge points, contrasting with D5, which showed significant relative abundances in eleven of the twelve months of study. Regarding the DW and TW samples, the results showed that the taxonomic diversity was not identical, but they shared similar taxa with the HWW. Genera such as *Bacteroides*, *Escherichia-Shigella*, *Prevotella*, *Aeromonas*, and *Pseudomonas* were identified with high relative abundances compared to HWW. Finally, the analysis of the diversity of genera in the WW samples showed one genus absent in the other water sources (*Enterococcus*), and *Prevotella* was absent. Common genera in the HWW, DW, and TW such as *Aeromonas*, *Escherichia-Shigella*, and *Faecalibacterium*, among others, were identified.

### 3.4. The Taxonomic Diversity in HWW Reveals Clinically Relevant Bacteria

**Figure 4:**
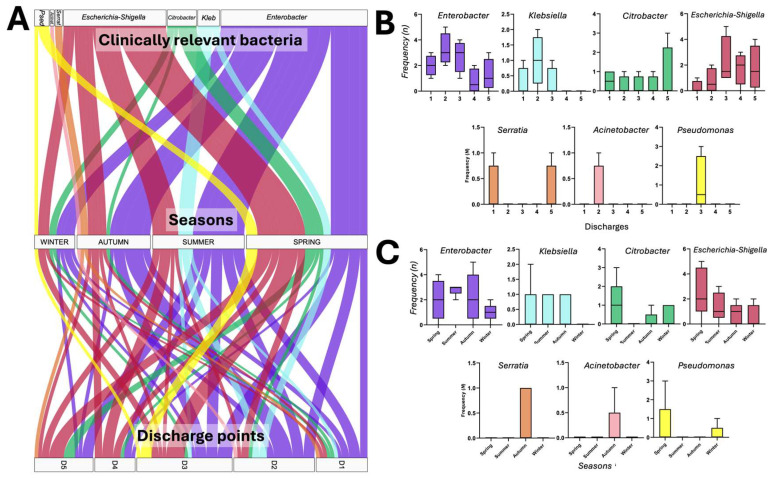
Characterisation of the clinically relevant bacteria (at genus level) released from the HWW of HJM. (**A**) Alluvial diagram of the release of clinically relevant bacteria by season and discharge; (**B**,**C**). ANOVA and Tukey’s post hoc tests on the release of clinically relevant bacteria by discharge point and seasonality. D1–D5: Discharge points.

During the analysis of the taxonomic diversity and relative abundance of bacterial taxa in the water sources, the genera of clinically relevant bacteria were identified (Figure 3B). This finding prompted a targeted analysis of this bacterial group to characterise the taxonomic diversity at the genus level by seasonality of release and by discharge point of this group. Figure 4A shows the release analysis of the clinically relevant bacteria classified by seasonality and discharge point (D1 to D5) of the HWW.

The results revealed that in 73.3% of the monitoring, clinically relevant bacteria were released from at least one discharge point during the study period, where the genus *Enterobacter* was the most frequent (33.3%), followed by *Escherichia-Shigella* (22.5%), *Citrobacter* (6.6%), *Klebsiella* (5%), *Pseudomonas* (3.3%), *Serratia* (1.6%), and *Acinetobacter* (0.8%). Alternatively, all discharge points released clinically relevant bacteria, where D3 bacteria were released 17 times (“17F” fortnights) during the year, followed by D2 (*n* = 15), D5 (*n* = 11), D1 (*n* = 10), and D4 (*n* = 8). Conversely, the taxonomic diversity and average relative abundance of the different water sources showed that there is active release of clinically relevant bacteria in all discharges, highlighting D4 with large amounts of faecal contamination (*Escherichia-Shigella*) and *Enterobacter* as the most abundant in D1, D2, D3, and D5 (Figure 4B,C).

### 3.5. Seasonal Detection of Antimicrobial Resistance Genes in HWW

**Figure 5:**
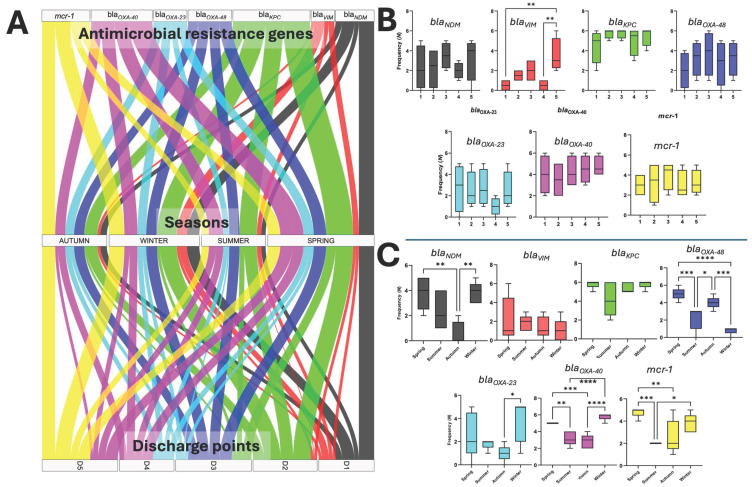
Antimicrobial resistance genes in HWW at HJM. (**A**) Alluvial plot of the detection of resistance genes by season and discharge point (D1 to D5); (**B**,**C**) ANOVA and Tukey’s post hoc tests in the detection of resistance genes by discharge and seasonality. Significance level: * (0.01), ** (0.001), *** (0.0001), and **** (0.00001).

The search for antimicrobial resistance genes to carbapenems by PCR in HWW was based on the local epidemiology of resistance in the ESKAPE bacteria of HJM. According to previous studies, metallo-β-lactamases (*bla_VIM_* and *bla_NDM_*) and serine β-lactamases (*bla_IMP_*, *bla_KPC_*, *bla_OXA-48_*, *bla_OXA-40_*, *and bla_OXA-23_*) are the ones circulating in this hospital. Conversely, although there is no record of colistin resistance due to the presence of the *mcr-1* gene in isolates of the ESKAPE bacteria in HJM, it was decided to include it in the resistance gene surveillance screening. The genes evaluated revealed that serine β-lactamases *bla_KPC_* and *bla_OXA-40_* were predominant in all discharges, with detection rates of 89.2 and 70%, respectively. Interestingly, the *mcr-1* gene was detected at 70%, followed by the carbapenemases *bla_OXA-48_* (50%), *bla_NDM_* (44.6%), *bla_OXA-23_* (38.3%), and *bla_VIM_* (26.6%) (Figure 5A). The metallo-β-lactamase *bla_IMP_* was not detected at any discharge point. In this context, Figure 5A shows the seasonal distribution of the seven antimicrobial resistance genes evaluated based on the season of the year, represented by spring, summer, autumn, and winter.

Finally, to determine whether there was a significant difference in the detection of resistance genes by discharge point and season, ANOVA and Tukey’s post hoc test were performed considering the means of the detection events of the seven genes during the study period (Figure 5B,C). The results revealed that there was no significant difference in six of the seven genes detected by discharge, with only the *bla_VIM_* gene showing a significant difference on D5. In contrast, the analysis by seasonality revealed that *bla_NDM_*, *bla_OXA-40_*, and *mcr-1* were the genes that showed statistical significance in their detection in the spring and autumn seasons (Figure 5C). In the case of *bla_OXA-48_* and *bla_OXA-23_*_,_ they were detected with a significantly higher frequency in spring and winter, respectively. Finally, no significant differences were identified in the detection of *bla_VIM_* and *bla_KPC_* in the seasons of the year evaluated.

### 3.6. Taxonomic Diversity and Average Relative Abundance of HWW, DW, TW, and WW

**Figure 6:**
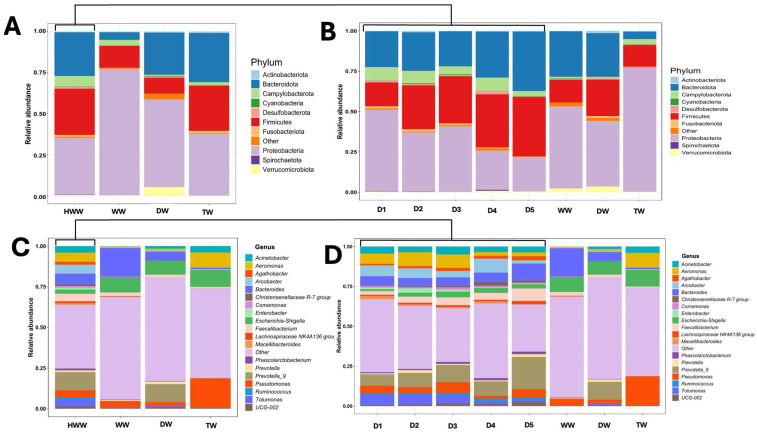
Relative abundance and average taxonomic diversity of HWW from HJM and other water sources. (**A**,**B**) Phylum. (**C**,**D**) Genus. HWW: hospital wastewater, DW: domestic wastewater, TW: treated wastewater, WW: water well of the Cutzamala and Nezahualcóyotl system, and D1 to D5: hospital discharge points.

The average relative abundance and taxonomic diversity at the five taxonomic levels (phylum, class, order, family, and genus) were analysed (Figure 6). For this purpose, a first taxonomic grouping by water origin was performed: HWW, DW, TW, and WW (Figure 6A,C), followed by a second grouping by HWW discharge: D1 to D5, DW, TW, and WW (Figure 6B,D). The results showed that the predominant phyla are Proteobacteria, Firmicutes, and Bacteroidetes in both groupings (Figure 6A). Interestingly, when sorting HWW by discharge point, Firmicutes are observed to progressively decrease from D1 to D5, while Campylobacterota become present in all discharges, along with Proteobacteria and Bacteroidetes as the predominant phyla (Figure 6B). Finally, the taxonomic composition of water samples categorised as DW, TW, and WW at this level showed no differences with HWW; however, Proteobacteria and Firmicutes were predominant. When analysing the taxonomic composition at the species level, it is observed that the HWW shows higher taxonomic complexity compared to the other water sources even though there is a group classified as “other” (Figure 6C). The analysis of the taxa at the species level by discharge shows the same level of complexity compared to the other water sources; however, the genera *Aeromonas*, *Bacteroides*, and *Faecalibacterium* and the strict anaerobe *Prevotella* stand out.

### 3.7. Alpha Diversity

**Figure 7:**
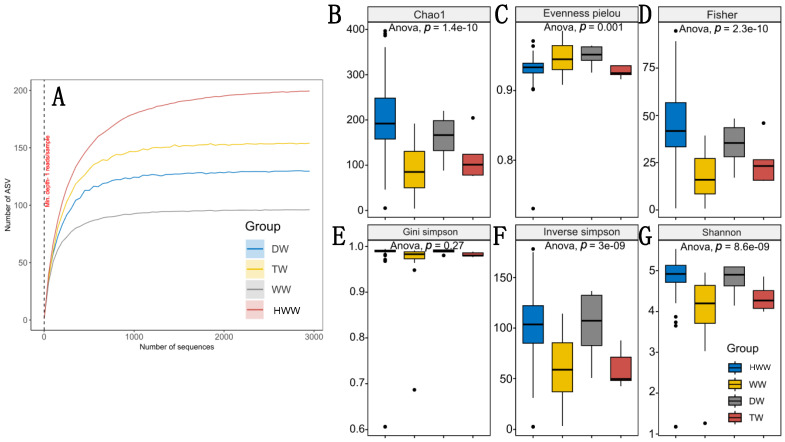
Alpha diversity of HWW from HJM. (**A**) Rarefaction curves, (**B**) the Chao1 index, (**C**) Pielou’s evenness, (**D**) Fisher, (**E**) Gini–Simpson, (**F**) inverse Simpson, and (**G**) Shannon. HWW: hospital wastewater, DW: domestic wastewater, TW: treated wastewater, WW: water well of the Cutzamala and Nezahualcóyotl system. Statistical significance *p* < 0.05.

The rarefaction curves of the amplicon sequence variants (ASVs) for each group of samples showed that HWW reached a higher richness score (about 200 ASVs) compared to the respective groups, with WW having the lowest richness (about 100 ASVs). The rarefaction curves of the samples from the four origins tended to asymptote, which indicated that the sequencing depth was sufficient to capture the microbial diversity present in each group for the alpha analysis (Figure 7A). ANOVA analysis of variance of alpha diversity using the first index “Chao1” between the four groups showed that HWW had significantly higher species richness compared to WW and TW (*p* = 1.4 × 10^−10^); the opposite was the case with DW, which showed no significant difference (Figure 7B). The evenness analysis of the bacterial species using the Evenness index (Pielou) revealed that HWW showed moderate evenness (about 0.9), with some variability with some species. This is because points out of range (below 0.8) were observed, where some samples of this group have dominance of certain species over others. In the case of the WW and DW samples, they show higher evenness (close to 1.0) compared to the HWW and TW, as they were the least even compared to all the groups (Figure 7C). The statistical significance (*p* = 0.001) confirms the observations described above for this index and shows that HWWs are complex with moderate equity but with high variability of species by origin and nature.

Regarding Fisher’s index, it was observed that HWWs have the highest species richness and diversity compared to other water types, suggesting that species richness varies among the samples analysed (*p* < 0.05). Statistical significance between groups shows that the observed differences in terms of species between samples are not random across this index (Figure 7D). The Gini–Simpson index showed two aspects; first, species diversity among the samples was high (values close to 1.0 in all cases) with variations among them but with no statistical significance (*p* = 0.27). This indicates that even though the biological nature of HWW is complex, the richness and heterogeneity are similar in the other water sources (Figure 7E). The index that allowed us to evaluate microbial diversity while considering species richness and equity among the four sample types (inverse Simpson) showed that in the HWW and DW, there is higher diversity compared to the WW and TW (Figure 7F), where the ANOVA test suggests that there are statistically significant differences (*p* = 3 × 10^−9^). Finally, the last index that allowed for the measuring of species richness and relative equity by sample type shows that HWW and DW showed consistently high values, which means that in these samples, there is higher species richness followed by WW and TW (Figure 7G).

### 3.8. Beta Diversity

**Figure 8:**
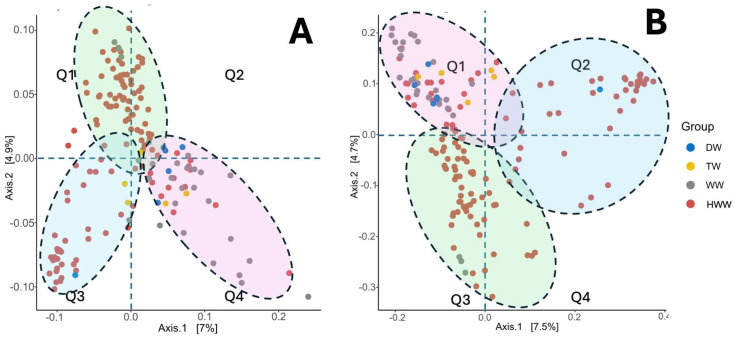
Beta diversity of HWW from HJM by weighted PCoA (**A**) and unweighted (**B**) UniFrac. HWW: hospital wastewater, DW: domestic wastewater, TW: treated wastewater, WW: water well of the Cutzamala and Nezahualcóyotl system. Statistical significance: *p* < 0.05.

Beta diversity analysis by unweighted PCoA and weighted UniFrac showed that the HWW bacterial populations are strongly separated from the rest of the populations of the other water sources (DW, TW, and WW), indicating that they have marked differences in composition (Figure 8A/Q1 and Figure 8B/Q3). Interestingly, two groups of HWW samples cluster in quadrants far from the main one (Figure 8A/Q3 and Figure 8B/Q2), suggesting that even though they are of hospital origin, they do not share compositional characteristics with the HWW groups described above. Conversely, the bacterial populations of DW, TW, and WW cluster together (Figure 8A/Q4 and Figure 8B/Q1), suggesting different compositional characteristics compared to HWW.

### 3.9. Influence of Physicochemical Factors on Bacterial Communities in HWW

The influence of the fourteen physicochemical factors on the bacterial communities of the HWW was evaluated by means of biplot analysis in principal components (PCA), complemented with PERMANOVA tests (multivariate permutational analysis of variance). This analysis considered environmental variables (the month and season of the year) as explanatory factors, with the purpose of identifying possible clustering patterns and trends related to specific environmental conditions and their impact on bacterial communities. The results of the PERMANOVA analysis showed statistical significance (*p* = 0.001), so it is concluded that there is an influence of these factors on the bacterial communities analysed by month and season of the year.

**Figure 9:**
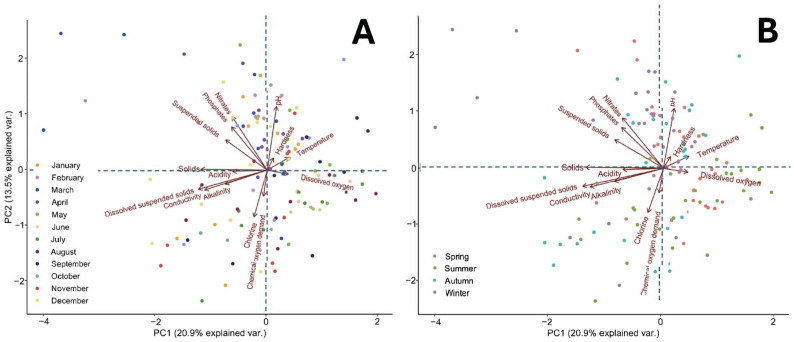
Seasonal influence of physicochemical factors on bacterial communities in hospital wastewater from HJM. (**A**). PCA based on the months of the year and (**B**) PCA by season.

As shown in Figure 9, principal component 1 (PC1) has 20.9% of the variation, while principal component 2 (PC2) has 13.5% of the variation, which means that between the two components, it contains 34.4% of the original physicochemical parameter data according to seasonality. It can be seen from the biplot in Figure 9A that dissolved solids, total solids, temperature, NO_3_^−^, and pH are the parameters that rescue the most data; therefore, they are the parameters that explain the changes in the model. This means that these physicochemical factors have the strongest relationship with species richness in the months of the year. Variables such as COD_2_ and pH are inversely proportional because they are antiparallel; similarly, solids and acidity are proportional. Alternatively, it is observed that suspended solids and hardness have a null correlation; a similar correlation is seen between chlorine and dissolved O_2_. The important data are those that are farthest apart, which represent the maximum and minimum of the parameters determined. The smaller the angle between the lines presenting them, the higher the correlation. The biplot in Figure 9B shows that suspended solids, total solids, NO_3_^−^, and pH are the parameters that retrieve the most data, which indicates that they are the parameters that explain the changes in the model. This means that these physicochemical factors have the strongest relationship with species richness over the seasons of the year, mostly in the winter and autumn seasons.

## 4. Discussion

Hospital wastewaters constitute an important focus of anthropogenic contamination, where bacteria of the ESKAPE group resistant to antibiotics are considered the most important due to the negative impact they have on Public Health and the environment [7,45]. Since Mexico has no legislation regulating the treatment of HWW before being released into the municipal sewage system, we speculate that there is active and seasonal release of medically important bacteria due to the healthcare activities of hospitals, where infectious diseases are the ones with the greatest impact on Public Health. Previous work by our working group has highlighted the microbiological problems associated with the presence of ESKAPE bacteria in the hospital environment, ranging from being outbreak-generating pathogens to bacteria that colonise medical devices due to their high capacity to form biofilms [40,41,42,46,47,48]. Therefore, the aim of the present work was to perform a seasonal analysis on the release of microbiological contamination, with emphasis on clinically relevant bacteria through HWW in one of the main tertiary-level hospitals in the country, HJM. This was addressed through a comprehensive analysis of the microbiome and antimicrobial resistance genes of epidemiological relevance at HJM while considering their relationship with key physicochemical variables of HWW. These variables have been recognised as important modulators of microbial diversity and abundance in aquatic environments [49,50]. As could be observed, this analysis not only allowed us to identify the total microbiological load at the five taxonomic levels in HWW (Figure 3 and Supplementary Material), but it also allowed us to understand how its composition may be influenced by some physicochemical variables at each of the HWW discharge points, with dissolved solids, total solids, temperature, NO_3_^−^, and pH being the most important (Figure 2 and Figure 9). The integration of the microbiological and physicochemical findings makes D4 stand out as the discharge point that contrasts with almost all the parameters analysed, suggesting that it is a key discharge point where hospital activities with the greatest impact on water quality are concentrated and where faecal waste predominates (Figure 1d). As can be seen in Figure 3 and Figure 6 and the Supplementary Material, the bacteriological richness of the HWW compared to the other water sources provided insights into how complex the taxonomic composition and relative bacterial abundance can be compared to waters from other sources (DW, TW, and WW). Both Alpha and beta diversities confirm the complexity and difference in microbiological composition of HWW with the other water sources for richness, species abundance, the evenness of distribution, the number of dominant species, and balance between richness and evenness (Figure 7 and Figure 8).

Several studies have examined the resistome and microbial communities in HWW by massive sequencing of the V3-V4 region, offering a useful framework to contextualise our findings. In a previous work, described marked differences between HWW and municipal wastewater in terms of both microbial taxonomic composition and resistance gene content. Their results showed a predominance of *Clostridiales*, *Lactobaciliales*, *Pseudomonadales*, *Aeromonadales*, *Actinomicetales*, and *Campylobacterales*, which were notably enriched in HWW [51]. In a subsequent longitudinal study, conducted a four-year surveillance effort in France, highlighting seasonal changes in both antimicrobial resistance gene (ARG) profiles and microbial community structures by V4 hypervariable region analysis. Genes related to aminoglycoside, b-lactam, and quinolone resistance were persistently enriched in HWW, while shifts in bacterial families such as *Clostridiales*, *Enterobacterales*, and *Pseudomonadales* suggested that healthcare activities influence the dynamic composition of the resistome and microbiota [50].

More recently, explored how hospital and urban wastewaters shape the resistome in biofilms, identifying *Acinetobacter*, *Pseudomonas*, and predominant genus as key taxa contributing to the persistence of ARGs [52]. A study in Portugal also drew attention to the taxonomic complexity of untreated hospital effluents by de novo massive sequencing. They reported high abundances of resistance genes against carbapenems and colistin, accompanied by dominant phyla including Proteobacteria, Actinobacteria, and Firmicutes [53]. In a context scope, a global meta-analysis and found that *bla_KPC_*, *bla_NDM_*, and *bla_OXA_* were among the most frequently detected carbapenemase genes in HWW across continents. These genes were often linked to genera like *Klebsiella*, *Acinetobacter*, and *Pseudomonas*, reinforcing their role as reservoirs of resistance [54]. In the Mexican context, analysed hospital effluents before and after treatment, noting that the persistent presence of *Escherichia coli Pseudomonas aeruginosa*, *Klebsiella pneumoniae*, *Enterococcus faecium*, *Enterobacter cloacae*, *Acinetobacter baumannii*, and *Staphylococcus aureus* and antimicrobial genes such as *bla_KPC_*, *bla_OXA_*, and *bla_NDM_* was consistently detected [6].

Our findings align with these observations, particularly regarding the dominance of clinically relevant bacteria taxa (the *Enterobacter*, *Escherichia-Shigella*, *Pseudomonas*, and *Acinetobacter* genus) and β-lactam resistance determinants such as *bla_KPC_*, *bla_OXA-40_*, and *bla_NDM_* (Figure 5 and Figure 6). However, a distinguishing feature of our study is the inclusion of seasonal variation and comparative sampling across untreated HWW, treated effluent, domestic sewage, and well water. This broader design not only underscores the complexity of hospital wastewater in terms of microbial and ARG diversity but also highlights its potential role in the environmental dissemination of clinically relevant pathogens in a country like Mexico, where HWW lacks specific treatment.

As mentioned, HWWs represent an environmental and sanitary challenge of great relevance due to their capacity to act as sources of contamination of bacteria of medical interest grouped under the acronym ESKAPE, which have been studied in Mexican hospitals through traditional culture [7,14,16]. Although their results are relevant, they have limitations such as the temporality of the study and the limited number of samples, which motivated us to perform an analysis aimed exclusively at elucidating the annual seasonal release of the total bacterial load and clinically relevant bacteria at each discharge point of HJM (Figure 1). It is important to highlight that massive sequencing of the V3-V4 region of the *16S rRNA* gene, although highly informative for bacterial profiling, does not always allow for precise identification at the species level, particularly for bacteria with high genetic similarity, such as the ESKAPE pathogens. In our study, we report the presence of genera that include clinically relevant species, but we acknowledge that these results represent an approximation rather than a confirmation at the species level. Interestingly, this work was carried out in parallel with a complementary study in the same hospital setting, where isolates were obtained and identified using classical culture methods, MALDI-TOF mass spectrometry, and resistance gene detection by PCR. This study confirmed the presence of ESKAPE group species, including *A. baumannii*, *K. pneumoniae*, the *Enterobacter* complex, and *P. aeruginosa* [19]. While both approaches differ in their methodology, the results are consistent and complementary. Together, they provide a more complete picture of the ESKAPE group’s presence and the potential risk in the HWW.

To our knowledge, only a research that has investigated the seasonal behaviour of the hospital and urban wastewater microbiome over a four-year period. This study was conducted in France and included longitudinal sampling from hospital discharge points and municipal wastewater treatment systems, providing valuable insights into temporal patterns in microbial composition and antimicrobial resistance [50]. The massive sequencing results of that study revealed dominant taxa at the order level in each water source, being *Clostridiales* for HWW and *Burkholderiales*, *Campylobacterales*, and *Pseudomonadales* in urban waters, respectively. Our results contrast with those of that study, where *Bacteroidales*, *Burkholderiales*, *Enterobacterales*, and *Campylobacterales* were the predominant phyla in all water samples, regardless of their origin (Supplementary Material). Therefore, the taxonomic diversity at higher resolution (genus level) of HWW, DW, TW, and WW was analysed for genetic fingerprinting of clinically relevant bacteria (Figure 3 and Figure 4).

The results contrast with other previously reported works; however, it should be noted that they highlight factors that influence this behaviour, such as the geographical location of the hospital, the type and capacity of care, water consumption per day/bed, medical services and procedures performed, and hygiene practices for infection control, among others [1,2,3,4]. Even though we speculated the existence of seasonal influence on the release of clinically relevant bacteria through Alluvial analysis (Figure 4A), ANOVA, and Tukey’s post hoc statistical analyses by discharge point and season, the results showed that their release is not influenced by these two variables (Figure 4B,C); however, bacteria of faecal origin such as *Enterobacter* and *Escherichia-Shigella* stand out as the most frequently identified in “D1, D2, D3, D5” and “D4”, respectively. D4 stands out as the discharge point where most hospital services converge (Figure 1d).

Within the ESKAPE microorganisms, which are classified as multidrug-resistant (MDR), extensively drug-resistant (XDR), and pandrug-resistant (PDR), they stand out as causative agents of various infections that are difficult to treat in HJM [40,41,42,46]. In this context, a relevant finding of this study was the identification of a seasonal behaviour in the release of β-lactam resistance genes in HWW (Figure 5C). Although techniques such as massive sequencing are widely used to characterise the resistome in this type of study, as they provide an overview of resistance mechanisms and genetic elements associated with their transfer [6,52,53], our experimental approach allowed us to analyse the resistance mechanisms circulating in our hospital from a local epidemiological perspective. This was performed to gain specific knowledge of the resistance genes that could possibly be released through HWW. The detection of the *mcr-1* gene at a high frequency in the five HJM discharges allows us to speculate that even though there are no reports of this circulating resistance mechanism in the ESKAPE pathogens detected in our hospital, its presence could be due to the high burden of faecal contamination, which has already been recognised as a source of microorganisms harbouring this polymyxin resistance mechanism [55,56]. The coincidence of resistance mechanisms identified in HWW and ESKAPE pathogens at HJM allows us to hypothesise the presence of a genetic interconnection and their potential release through the hospital’s care activities.

To clarify this hypothesis, we aimed at studying the HWW of HJM with a traditional seasonal culture approach to identify antibiotic resistant microbiological loads, such as those used in the antimicrobial therapy of our hospital, for the targeted search of the resistance mechanisms identified in the present work, together with molecular tools of clonality in ESKAPE pathogens [19]. The evidence presented in this work is oriented towards the creation of strategies such as programmes that include the efficient management of HWW and, ideally, the implementation of HWW treatment plants, without losing sight of the fact that even though this is one of the best strategies to mitigate the associated risks, there is research that shows the persistence of bacterial pathogens post-treatment and resistance genes in HWW [6].

## 5. Conclusions

Hospital wastewaters represent an environmental and sanitary challenge of great relevance due to their capacity to act as sources of chemical and microbiological contamination, so the findings of our study complement previous research by our working group on clinically relevant bacteria as the generator of in-hospital problems due to their presence in HWW together with antimicrobial resistance genes. This research aims to understand the risks associated with HWW to lay the groundwork for the development of mitigation strategies that address both microbiological and physicochemical aspects in an integrated manner. This knowledge generated should be used to address the challenges posed by hospital contamination in the current context of antimicrobial resistance and the environmental impact due to the risk they pose.

## Figures and Tables

**Table 1:** Primers used in this study.

Primer	Molecular Target	Sequence (5′→3′)	Size (bp)	Reference
*IMP-F*	*bla_IMP_*	TTGACACTCCATTTACDG	139	[43]
*IMP-R*		GATYGAGAATTAAGCCACYCT		
*VIM-F*	*bla_VIM_*	GATGGTGTTTGGTCGCATA	390	
*VIM-R*		CGAATGCGCAGCACCAG		
*KPC-F*	*bla_KPC_*	CATTCAAGGGCTTTCTTGCTGC	538	
*KPC-R*		ACGACGGCATAGTCATTTGC		
*OXA-48F*	*bla_OXA-48_*	GCACTTCTTTTGTGATGGC	281	
*OXA-48R*		GAGCACTTCTTTTGTGATGGC		
SHV-F	*bla_SHV_*	AGCCGCTTGAGCAAATTAAAC	713	
SHV-R		ATCCCGCAGATAAATCACCAC		
CTX-M-1-F	*bla_CTX_*	TTAGGAARTGTGCCGCTGYA	688	
CTX-M-1-R		CGATATCGTTGGTGGTRCCAT		
*NDM-F*	*bla_NDM_*	GGTTTGGCGAT CTGGTTTTC	621	[44]
*NDM-R*		CGGAATGGCTCATCACGATC		

## Data Availability

The databases of sequences are registered at NCBI with the BioProject ID PRJNA1268496 at http://www.ncbi.nlm.nih.gov/bioproject/1268496 (accessed date on 1 June 2025). Relative abundance and average taxonomic diversity analysis of HWW from Hospital Juárez de México and other water sources (four taxonomic level additional) are available in Bello-López and Juan Manuel (2025), Mendeley Data, V1, URL ID: https://data.mendeley.com/datasets/5n376rhxx9 (accessed date on 1 June 2025).

## Supplementary Materials

The following supporting information can be downloaded at: https://www.mdpi.com/article/10.3390/microorganisms13061316/s1, Figure S1: Variation in taxonomic diversity and relative abundance (at class level) of bacterial communities released through the HWW of the Juárez Hospital of Mexico. WWH: hospital wastewater, DW: domestic wastewater, TW: treated wastewater and WW: water well of the Cutzamala and Nezahualyotl systems. Figure S2: Variation in taxonomic diversity and relative abundance (at order level) of bacterial communities released through the HWW of the Juárez Hospital of Mexico. WWH: hospital wastewater, DW: domestic wastewater, TW: treated wastewater and WW: water well of the Cutzamala and Nezahualyotl systems. Figure S3: Variation in taxonomic diversity and relative abundance (at family level) of bacterial communities released through the HWW of the Juárez Hospital of Mexico. WWH: hospital wastewater, DW: domestic wastewater, TW: treated wastewater and WW: water well of the Cutzamala and Nezahualyotl systems. Figure S4: Variation in taxonomic diversity and relative abundance (at species level) of bacterial communities released through the HWW of the Juárez Hospital of Mexico. WWH: hospital wastewater, DW: domestic wastewater, TW: treated wastewater and WW: water well of the Cutzamala and Nezahualyotl systems.
