# Supplementary material for: Profiling of Bacterial Communities of Hospital Wastewater Reveals Clinically Relevant Genera and Antimicrobial Resistance Genes
Source: Microorganisms. 2025 Jun 5;13(6):1316. doi: 10.3390/microorganisms13061316 (PMC12195092; doi:10.3390/microorganisms13061316)
Supplement: Supplementary file 1 [file microorganisms-13-01316-s001.zip › microorganisms-3612916-supplementary.pdf]

Supplementary

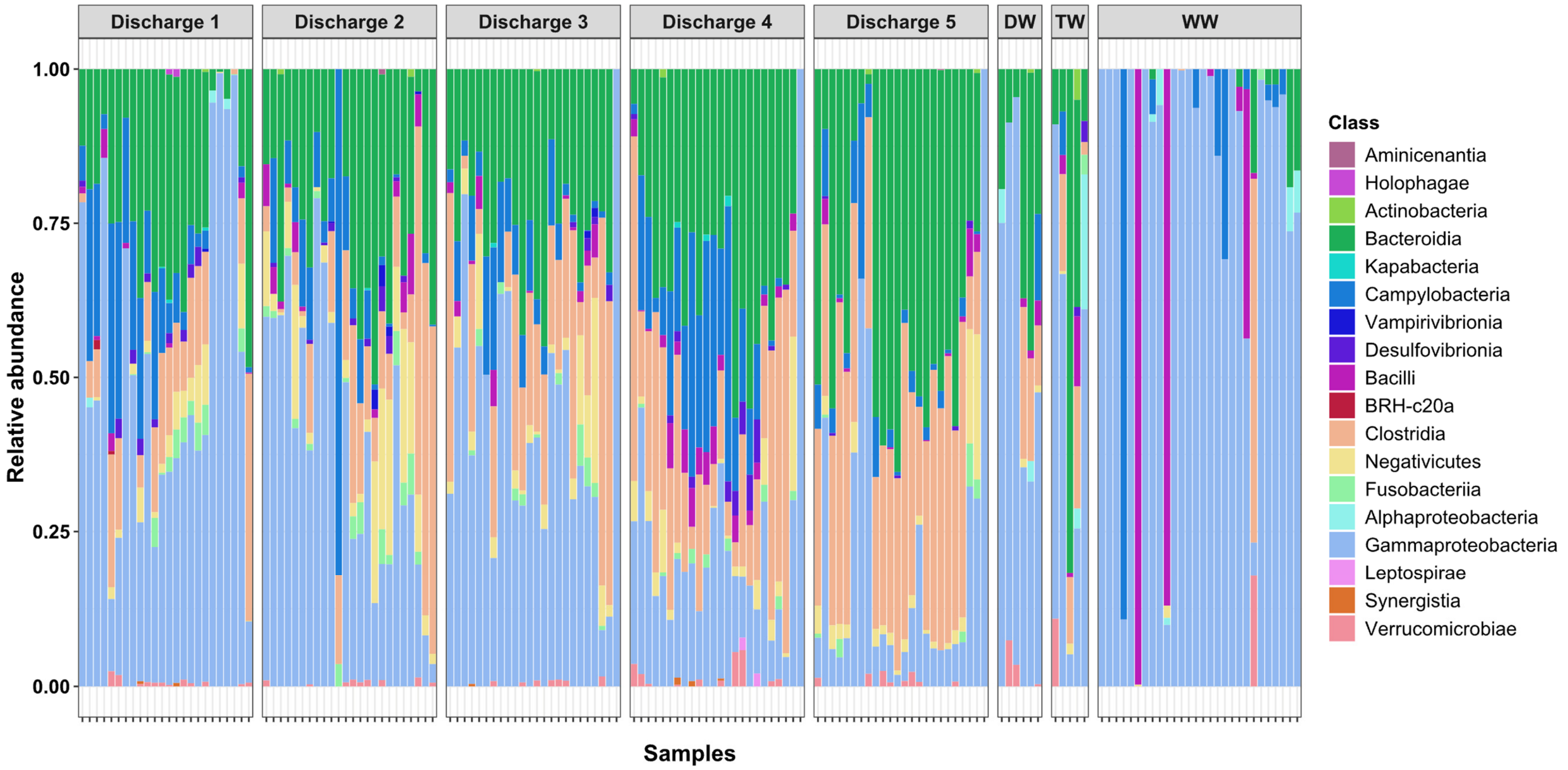

Supplementary Figure S1: Variation in taxonomic diversity and relative abundance (at class level) of bacterial communities released through the HWW of the Juárez Hospital of Mexico. WWH: hospital wastewater, DW: domestic wastewater, TW: treated wastewater and WW: water well of the Cutzamala and Nezahualyotl systems.

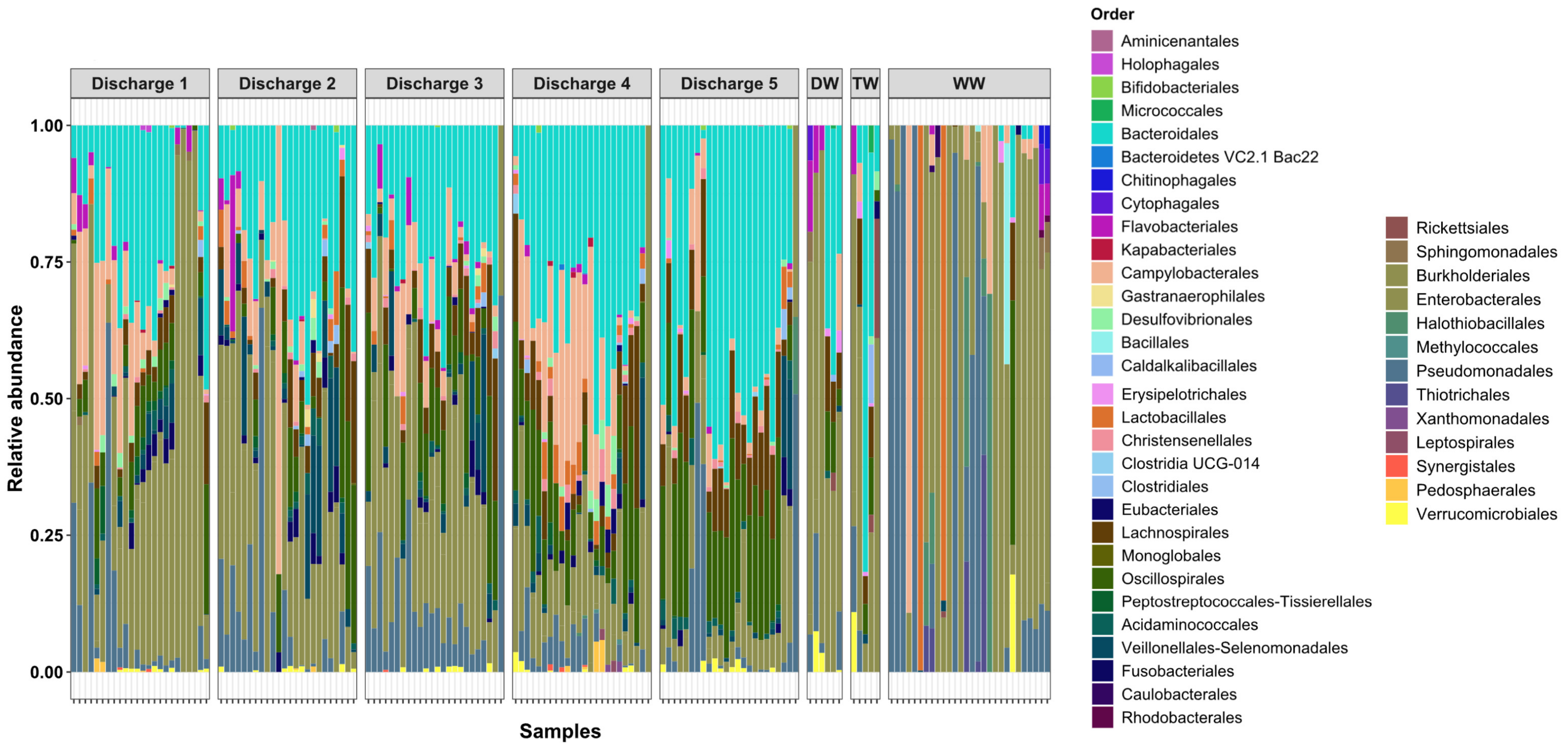

Supplementary Figure S2: Variation in taxonomic diversity and relative abundance (at order level) of bacterial communities released through the HWW of the Juárez Hospital of Mexico. WWH: hospital wastewater, DW: domestic wastewater, TW: treated wastewater and WW: water well of the Cutzamala and Nezahualyotl systems.

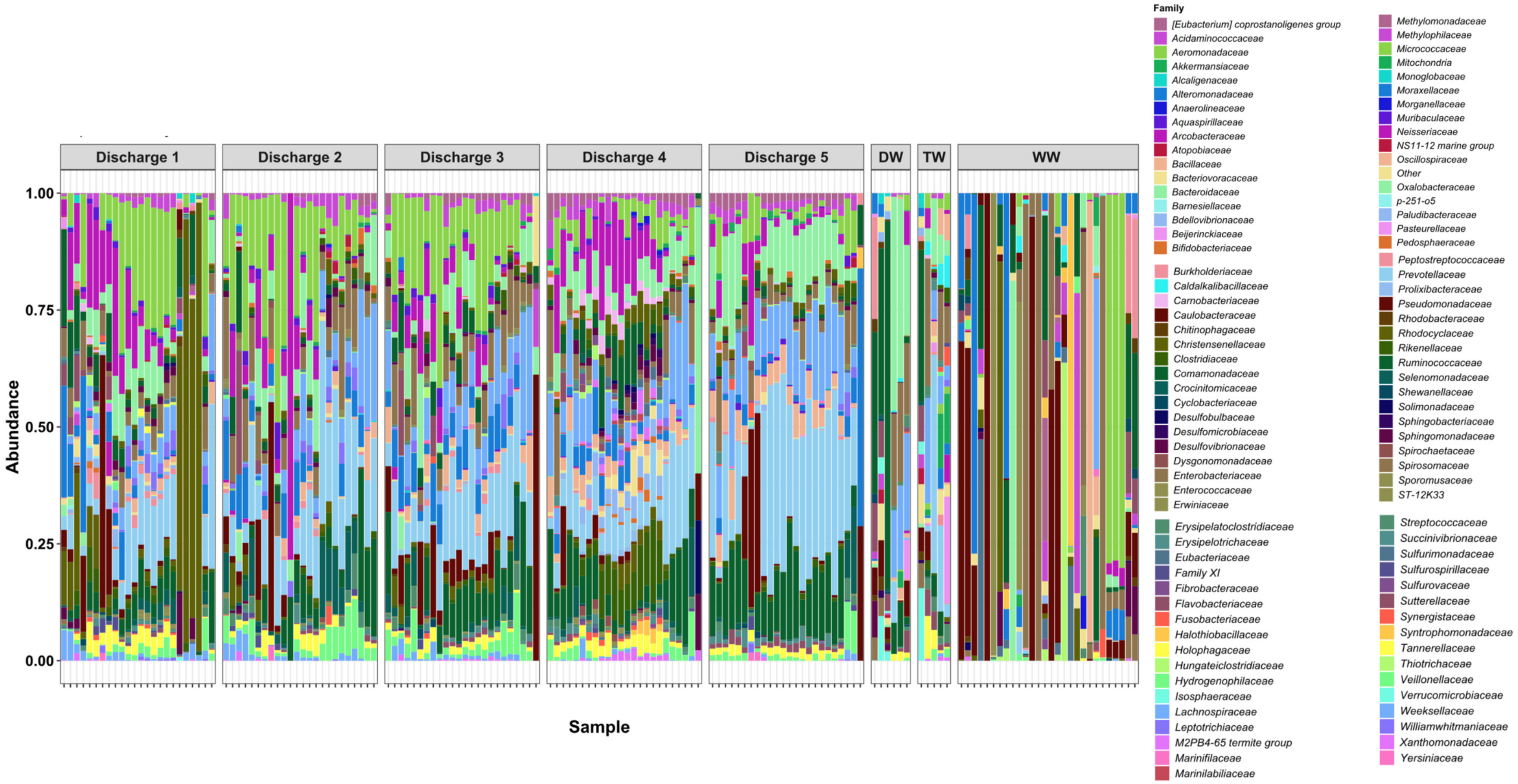

Supplementary Figure S3: Variation in taxonomic diversity and relative abundance (at family level) of bacterial communities released through the HWW of the Juárez Hospital of Mexico. WWH: hospital wastewater, DW: domestic wastewater, TW: treated wastewater and WW: water well of the Cutzamala and Nezahualyotl systems.

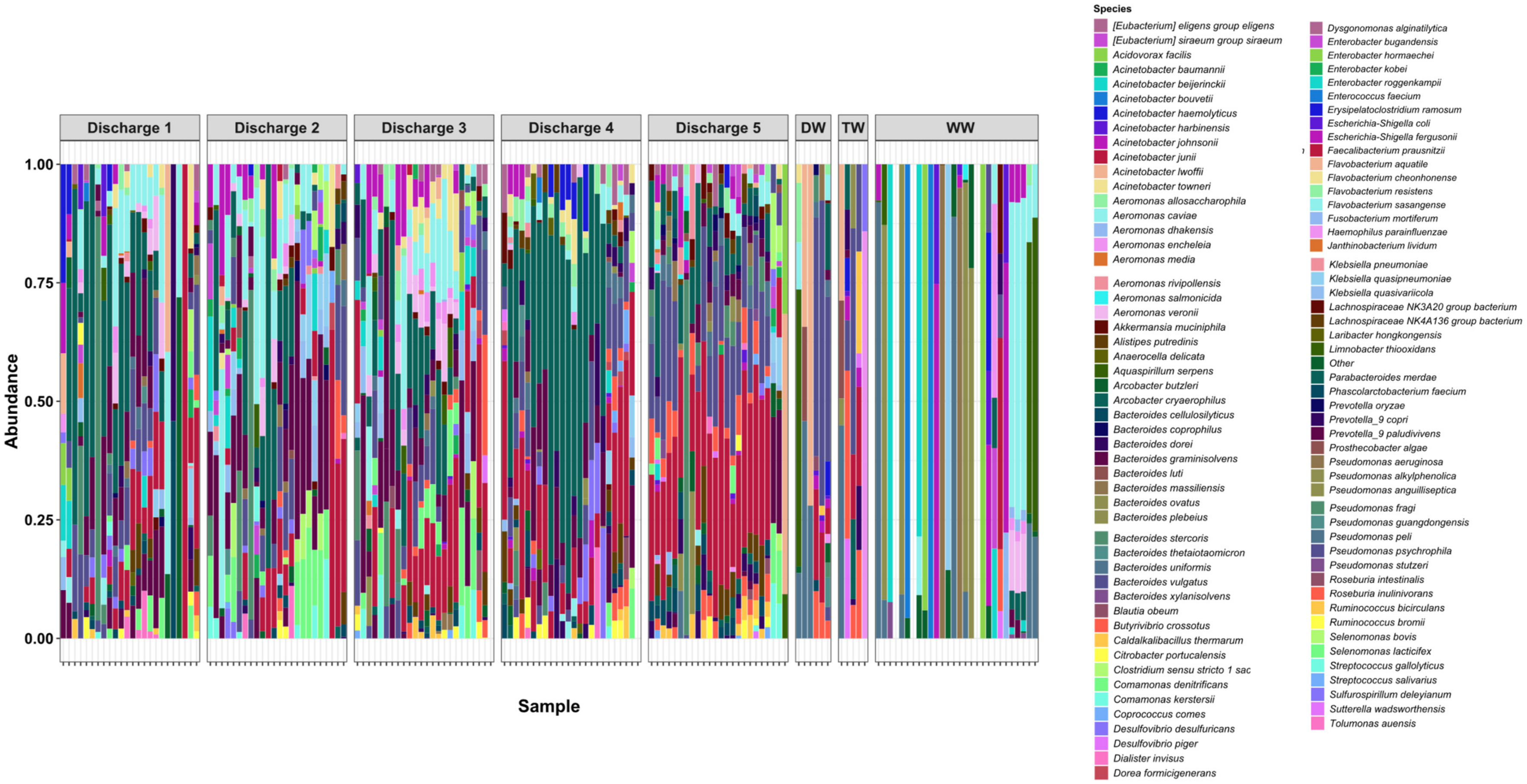

Supplementary Figure S4: Variation in taxonomic diversity and relative abundance (at species level) of bacterial communities released through the HWW of the Juárez Hospital of Mexico. WWH: hospital wastewater, DW: domestic wastewater, TW: treated wastewater and WW: water well of the Cutzamala and Nezahualyotl systems.
